# Diastolic Heart Failure: A Concise Review

**DOI:** 10.4021/jocmr1532w

**Published:** 2013-08-05

**Authors:** Fahad Aziz, Luqman-Arafath TK, Chijioke Enweluzo, Simanta Dutta, Misbah Zaeem

**Affiliations:** a Department of Internal Medicine, Section on Hospital Medicine, Medical Center Boulevard, Winston Salem, NC 27157, USA

**Keywords:** Diastolic Heart Failure, Pathogenesis, Treatment

## Abstract

The concept of “diastolic” heart failure grew out of the observation that many patients who have the symptoms and signs of heart failure had an apparently normal left ventricular (LV) ejection fraction. Thus it was assumed that since systolic function was “preserved” the problem must lie in diastole, although it is not clear by whom or when this assumption was made. Nevertheless, many guidelines followed on how to diagnose “diastolic” heart failure backed up by indicators of diastolic dysfunction derived from Doppler echoardiography. Diastolic heart failure is associated with a lower annual mortality rate of approximately 8% as compared to annual mortality of 19% in heart failure with systolic dysfunction, however, morbidity rate can be substantial. Thus, diastolic heart failure is an important clinical disorder mainly seen in the elderly patients with hypertensive heart disease. Early recognition and appropriate therapy of diastolic dysfunction is advisable to prevent further progression to diastolic heart failure and death. There is no specific therapy to improve LV diastolic function directly. Medical therapy of diastolic dysfunction is often empirical and lacks clear-cut pathophysiologic concepts. Nevertheless, there is growing evidence that calcium channel blockers, beta-blockers, ACE-inhibitors and ARB as well as nitric oxide donors can be beneficial. Treatment of the underlying disease is currently the most important therapeutic approach.

## Introduction

Heart failure is most commonly associated with impaired LV systolic function. However, as many as 30-40% of all patients with typical symptoms of congestive heart failure, have a normal or slightly reduced ejection fraction. In these patients, diastolic dysfunction is implicated as a major contributor, if not the primary cause of congestive heart failure [1, 2]. The syndrome of clinical heart failure with normal left ventricular systolic function in the absence of cardiac valvular lesions is often referred to as diastolic heart failure (DHF). However, the diagnosis of isolated diastolic dysfunction as a cause of heart failure remains controversial.

Primary diastolic dysfunction is typically seen in patients with hypertension and or restrictive cardiomyopathy but can also occur in a variety of other clinical disorders and has a particularly high prevalence in the elderly population [3, 4]. In the Helsinki Ageing Study, 51% of patients aged 75 - 86 years with clinical heart failure were thought to have DHF [5].

Although diastolic heart failure is common in clinical practice worldwide, its existence has been questioned for several reasons [6-8]. Firstly, investigators have questioned whether these patients truly have heart failure or if they actually have conditions such as obesity or pulmonary disease that can mimic heart failure [9]. In a study of a direct access echocardiography service, Caruana et al concluded that most patients with suspected heart failure and preserved systolic function were inappropriately labeled as having diastolic heart failure, and in fact had other factors including lung disease causing their symptoms [9].

Doubts regarding diastolic heart failure are cast especially because the diagnosis of heart failure is partly clinical and prone to error. When the left ventricular ejection fraction is low the diagnosis of heart failure is seldom questioned-clinicians seem more willing to accept a diagnosis of systolic heart failure. Fortunately the advent of biomarkers such as plasma B-type natriuretic peptides should help confirm the presence of heart failure in patients with suspected diastolic heart failure.

A second area of controversy is that while investigators may agree that some patients with heart failure do have a normal ejection fraction, they doubt if the underlying mechanism is truly left ventricular diastolic dysfunction, as implied by the term diastolic heart failure. Some of these patients have subtle abnormalities of systolic function (although the ventricular ejection fraction is normal). In some case series the relations between left ventricular pressure and volume on cardiac catheterization do not conform to a classical pattern of diastolic dysfunction [10]. The evidence base for the diagnosis and treatment of diastolic heart failure has lagged behind systolic heart failure mainly secondary to the foregoing points.

## Patho-physiology of Diastolic Heart Failure

The patho-physiology of diastolic heart failure is characterized by a low cardiac output that results typically from a ventricle that has thick walls but a small cavity (increased left ventricular mass/volume ratio) [11]. When the left ventricle is stiff, it relaxes slowly in early diastole and offers greater resistance to filling in late diastole, so the diastolic pressures are elevated. The low cardiac output manifests as fatigue, while the higher end diastolic pressure is transmitted backwards through the valve- less pulmonary veins to the pulmonary capillaries, resulting in exertional dyspnea. These patho-physiological abnormalities trigger neurohormonal activation as happens in systolic heart failure. Symptoms may be unmasked by exercise because, unlike normal people, patients with diastolic heart failure are unable to augment their stroke volume by increasing their left ventricular end diastolic volume (Frank-Starling mechanism). These patients often have an exaggerated response of systolic blood pressure to exercise. Mechanisms contributing to abnormal left ventricular diastolic properties include stiff large arteries, hypertension, myocardial ischemia, diabetes, and intrinsic myocardial changes with or without associated hypertrophy [11].

## Specificity of Symptoms and Signs

The symptoms (dyspnea, fatigue, exercise intolerance), signs (jugular venous distension, pulmonary rales, peripheral edema), and radiographic evidence (pulmonary vascular redistribution, interstitial edema, pleural effusions) of heart failure (HF) occur with equal frequency in patients with diastolic heart failure when compared with patients with systolic heart failure [12]. Therefore, the history, physical exam, and chest X-ray are not specific enough to differentiate the two entities. Taken together, however, they are specific enough to detect the presence of HF. The clinician’s ability to use this clinical evidence in diagnosing HF has been questioned [9, 13]. Curiously, this uncertainty has only been discussed with reference to DHF and has not been questioned in patients with SHF. Characteristics of SHF and DHF are compared in Table 1. It has been argued that some patients with DHF have symptoms and signs for which an explanation other than HF can be identified [9, 13]. For example, older patients who are significantly de-conditioned may have symptoms of fatigue that may not be caused by HF. Like wise, patients with chronic lung disease may have dyspnea that is not caused by HF. The advent of widely available assays of brain natriuretic peptide and other new noninvasive techniques may make this problem of accurately diagnosing HF less challenging. This challenge can also be overcome by using the strict, predefined, and validated criteria that include symptoms and signs of HF such as those used by Smith et al [14] and others [8].

**Table 1:** Characteristics of Systolic vs Diastolic Heart failure

Characteristics	Systolic heart failure	Diastolic heart Failure
Systolic function		
Ejection fraction	↓↓	N or ↑
Stroke volume	↓	N or ↓
Contractility	↓↓	↓
Diastolic Function		
LV end diastolic pressure	↑ or ↑↑	↑↑ or ↑
Relaxing time constant	↑ or ↑↑	↑↑ or ↑
Filling Rate	↓ or ↑ or N	↓ or ↑ or N
Chamber stiffness	↓	↑↑
Myocardial Stiffness	N or ↑	↑
Mortality		
At 6 months	↑↑	↑
At 5 years	↑↑	↑

## Co-existence of Systolic and Diastolic Dysfunction

It is now clearly recognized that all patients with HF have abnormalities in both systolic and diastolic function. However, the coexistence of these functional abnormalities does not necessarily mean that SHF and DHF are part of a single continuum. There are significant differences between SHF and DHF that justify their separation into two different groups.

There is no doubt that a normal EF does not necessarily indicate the presence of normal myocardial or even ventricular contractility [15, 16]. Therefore, patients with HF and preserved EF may have relatively small but detectable abnormalities in systolic function. For this reason the terms HF with preserved systolic function should not be used. However, there are no convincing data to support the idea that abnormalities in contractility are responsible for the symptoms and signs of HF, or the patho-physiologic remodeling that is seen in patients with DHF. Patients with DHF commonly have concentric remodeling characterized by normal LV volume, increased LV mass, increased wall thickness, decreased volume-to-mass ratio, and increased chamber and myocardial stiffness. There is no conceptual framework to support the notion that abnormal contractility contributes causally to this concentric remodeling. Therefore, the presence of abnormal indices of contractility in patients with DHF does not negate the fact that the predominant abnormality is diastolic dysfunction, nor does it support the idea that this abnormal contractility is the mechanism responsible for the development of DHF. Thus, HF in patients with DHF is caused by a predominate (although not isolated) abnormality in diastolic function.

## Diagnosis of Diastolic Heart Failure

The diagnosis of diastolic heart failure requires three conditions to be simultaneously satisfied [17]: 1), Presence of signs and symptoms of heart failure; 2), Presence of normal or only slightly reduced LV ejection fraction (EF. 50%) a; 3) Presence of increased diastolic pressure or impaired filling caused by delayed iso-volumic relaxation or elevated stiffness.

Patients with shortness of breath on exertion, pulmonary rales, or gallop sound but with near normal systolic function are usually diagnosed with DHF [18].

### Non-invasive assessment of diastolic function

Several non-invasive techniques have been used for assessing diastolic function in patients with coronary, valvular or myocardial heart disease. The most commonly used methods are 2D- and Doppler-echocardiography, Doppler-tissue imaging, radionuclide ventriculography, MR myocardial tagging and MR imaging [19].

#### Echocardiography

M-mode echocardiography has been used to assess the dimensional changes and its first derivates during diastolic filling. The presence of LV myocardial fibers oriented in the longitudinal direction and located in the trabecular, subendocardial, and papillary muscle regions has led to an increasing interest in echocardiographic assessment of the LV long axis function. As displacement of the atrioventricular plane towards the apex is a direct reflection of longitudinal fiber contraction, its measurement by M mode echocardiography provides additional information regarding global and regional systolic and diastolic function. As shown by Henein and Gibson, delayed onset of lengthening can effectively suppress early diastolic trans-mitral flow, even though the minor axis increases and mitral cusps separate normally [20].

During the last 2 decades Doppler-echocardiography has emerged as an important clinical tool providing reliable and useful data on diastolic performance. Three different approaches are routinely used in the assessment of diastolic dysfunction: measurement of trans-mitral and pulmonary venous flow as well as intra-ventricular filling patterns (Doppler flow propagation) [19].

The trans-mitral velocity pattern remains the starting point of echocardiographic assessment of LV diastolic function; since it is easy to acquire and can rapidly categorize patients with normal or abnormal diastolic function by E/A ratio (early to late filling velocity) [20, 21]. In healthy young individuals, most diastolic filling occurs in early diastole so that the E/A ratio is ( 1. When relaxation is impaired, early diastolic filling decreases progressively and a vigorous compensatory atrial contraction (‘atrial kick’) occurs. The results in a reversed E / A ratio, increased deceleration time, and increased iso-volumic relaxation time [21]. With disease progression LV compliance becomes reduced and filling pressures begin to increase leading to compensatory augmentation of left atrial pressure with increase in early filling despite impaired relaxation, so that filling pattern looks relatively normal (‘pseudo-normalization’ pattern ( E/A ( 1) [21]. Finally, in patients with severe decrease in LV compliance, left atrial pressure is markedly elevated and compensates with vigorous early diastolic filling for impaired relaxation. This ‘restrictive’ filling pattern (E/A ( 1) is consistent with an abnormal rise in LV pressure and an abrupt deceleration of flow with little additional filling during mid-diastole and atrial contraction. In extreme cases the LV pressure rise overshoots left atrial pressure so that diastolic mitral regurgitation in mid diastole may be seen.

Color Doppler M-mode provides a unique window into the fluid dynamics of flow across the mitral valve. The speed of propagation is enhanced with rapid relaxation and LV suction. Clinical and experimental studies have demonstrated that the inverse correlation to t is relatively independent of left atrial pressure [22]. Furthermore, combined evaluation of flow propagation velocity and early diastolic annular velocity can be used for estimation of filling pressure [23].

Doppler tissue imaging yields information on intra-myocardial velocity, providing a unique insight into LV mechanics during iso-volumic contraction and relaxation. In normal persons the mitral annular motion is almost a mirror image of the trans-mitral flow pattern, but in patients with pseudo normal or restrictive filling pattern, annular motion is abnormally low, implying that it is relatively independent of preload [24, 25].

It has been shown that relaxation velocities in the myocardium are inversely correlated with t, so that a non-invasively calculation of the time constant of relaxation seems to be possible [26, 27]. Through the integrated use of Doppler echocardiography and Doppler tissue imaging, it is possible to obtain a fairly precise picture of LV diastolic function [28]. However, atrial fibrillation or frequent ectopic beats are the major limitation of these techniques. To overcome this problem, averaging of several heart cycles with similar RR intervals has been proposed.

#### Magnetic resonance imaging

This technique has been shown to be of considerable use in the morphologic assessment of the heart, but functional assessment can also be obtained. However, their clinical relevance remains to be demonstrated [29]. Additional information may be gained from newer techniques such as magnetic resonance myocardial tagging, which allows the labeling of specific myocardial regions [30]. From these tags the rotational and translational motion of the left ventricle can be determined, which is characterized by a systolic wringing motion followed by a rapid diastolic untwisting [31]. This untwisting motion is directly related to relaxation and may be used as a measure of the rate and completeness of relaxation as well as an estimate of early diastolic filling.

#### Radionuclide angiography

This technique may be used to study the rapid filling phase of diastole, the duration of the iso-volumic relaxation phase, the relative contribution of rapid filling to total diastolic filling, and the relation between regional non-uniformity of left ventricular function and global filling properties [32-34]. However, radionuclide angiography does not permit assessment of the left atrial - left ventricular pressure gradient or the simultaneous evaluation of changes in left ventricular pressure and volume during relaxation and filling. Therefore, complete clinical interpretation of ‘abnormal’ left ventricular filling indexes, or changes in these indexes after interventions, is not possible. Despite the inherent limitations of noninvasive assessment of left ventricular diastolic function, radionuclide evaluation of left ventricular filling may provide clinically useful insights [35].

### Invasive assessment of diastolic function

Cardiac catheterization with simultaneous pressure and volume measurements is the ‘gold’ standard for assessing LV diastolic function. Prerequisites are high-fidelity pressure recordings with simultaneous angio- or echocardiography or the use of the conductance technique. The rate of LV relaxation, rate and timing of diastolic filling as well as myocardial and chamber stiffness can be determined [36].

## Management of Diastolic Dysfunction

Despite the large number of individuals suffering from diastolic dysfunction, there is a paucity of recommendations available to help guide medical therapy in these patients. The guidelines for the European Society of Hypertension (ESH) only briefly touch on the subject of diastolic dysfunction and offer no specific recommendations for its management, commenting that little information is available comparing the effects of different antihypertensive agents on diastolic dysfunction [37]. The Joint National Committee on Prevention, Detection, Evaluation, and Treatment of High Blood Pressure also discussed the high prevalence of impaired LV relaxation in hypertensive patients, but had no specific recommendations on its management [38]. Thus, for the moment, there are no definitive guidelines for treating diastolic dysfunction.

Diastolic dysfunction may be present for several years before any symptoms occur and may represent the first phase of diastolic heart failure. Thus, it is important to detect diastolic dysfunction early and to start treatment before irreversible structural alterations and systolic dysfunction have occurred.

Four treatment goals have been advocated for the therapy of diastolic dysfunction: 1), Reduction of central blood volume (diuretics); 2), Improvement of LV relaxation (calcium channel blockers or ACE inhibitors); 3), Regression of LV hypertrophy (decrease in wall thickness and removal of excess collagen by ACE inhibitors, ARB); 4), Maintenance of atrial contraction and control of heart rate (beta-blockers and antiarrhythmic).

### Diuretics

Diuretics are effective in reducing pulmonary congestion in patients with diastolic dysfunction, shifting the pressure-volume relation downwards. However, their positive effect on LV chamber stiffness is indirect, caused particularly by a reduction in systemic blood volume and the lowering of right atrial blood volume with a decrease in pericardial constraint. However, they must be used judiciously because the volume sensitivity of patients with diastolic dysfunction bears the risk that excessive diuresis results in sudden drop of stroke volume [39]. The steep pressure - volume relationship, has explained volume sensitivity when stroke volume can be maintained only with a high filling pressure. A small decrease in diastolic filling pressure could result in a drop in stroke volume, thus, cardiac output.

### Beta-blockers

Beta-blockers have long been theorized to provide benefit to patients with diastolic dysfunction. By slowing the heart rate to allow more time for LV filling and lowering blood pressure to improve LV compliance, beta- blockers can improve diastolic function [1]. Tachycardia has also been shown to be detrimental to patients with diastolic dysfunction because the increased heart rate shortens the diastolic filling period resulting in less time for LV relaxation and worse compliance [40]. These concepts have been validated in the SILVHIA trial, which found that after 48 weeks of treatment with Atenolol, patients with diastolic dysfunction and hypertension had improvements in indices of diastolic function [41]. Another smaller study also showed that after 6 months of treatment with Atenolol or Nebivolol, hypertensive patients with diastolic dysfunction had decreased LV mass and an improved E/A ratio [42]. There is conflicting data regarding the clinical benefits of these improved diastolic filling parameters. In subgroup analysis of the patients with DHF, Nebivolol did not show a reduction in all-cause mortality or cardiovascular hospital admissions at 21 months [43]. Two small studies showed clinical benefits with beta-blockers in DHF. The first examined the impact of Propranolol on 158 patients with heart failure and ejection fraction ( 40% [44]. At 32 months, patients receiving Propranolol had a 35% reduction in total mortality and a 37% reduction in the composite of total mortality or nonfatal myocardial infarction. The second trial showing the benefit of beta blockade in DHF consisted of 40 patients with mild or moderate heart failure and EF > 45% [45].

### Calcium channel blockers

Calcium channel blockade is thought to improve diastolic function by several mechanisms, including attenuating calcium homeostasis, reducing blood pressure, promoting LV hypertrophy regression, and slowing the heart rate [46]. Benefits of calcium channel blockade on diastolic function have been examined in a few studies. In the ELVERA trial, the use of Amlodipine in hypertensive patients led to a reduction in LV mass and improved diastolic function at 2-year follow-up [47]. In a sub-study of the Anglo- Scandinavian Cardiac Outcomes Trial (ASCOT), hypertensive patients with diastolic dysfunction were treated with Amlodipine and had improved diastolic filling parameters at 1-year follow-up [48]. Calcium blockers of the verapamil type are first line drugs in patients with hypertrophic cardiomyopathy due to their beneficial effect on relaxation and diastolic filling, which are often severely abnormal in these patients [49].

### ACE-inhibitors and ARB

ACE inhibitors and ARB’s show not only an effect on blood pressure but elicit a direct effect on the myocardium via the local renin-angiotensin system. These effects are essential for the regression of LV hypertrophy, and the improvement in the elastic properties of the myocardium [50, 51]. Several trials have documented that LV hypertrophy is more effectively reduced by ACE inhibitors than by any other antihypertensive drugs, suggesting an effect on myocardial structure beyond that provided by the reduction of pressure overload. Recent data suggest that ARBs have similar effects on LV mass and structure such as ACE inhibitors. In a direct comparative trial of ARBs and beta-blockers, it was demonstrated that despite similar reductions in blood pressure, regression of LV hypertrophy with Irbesartan was greater than those attained with Atenolol [52].

### Digitalis

Digitalis should not be used in patients with diastolic dysfunction, except in those with atrial fibrillation. When atrial fibrillation occurs, restoration of sinus rhythm is mandatory and is best achieved with electric conversion. For prophylaxis of recurrence, beta-blockers, especially sotalol, which exerts a class III antiarrhythmic effect and amiodarone are the most suitable. 
